# The Impact of Hyperoxia on Outcome of Patients Treated with Noninvasive Respiratory Support

**DOI:** 10.1155/2020/3953280

**Published:** 2020-05-06

**Authors:** Seyhan Pala Cifci, Yasemin Urcan Tapan, Bengu Turemis Erkul, Yusuf Savran, Bilgin Comert

**Affiliations:** ^1^Department of Internal Medicine, Dokuz Eylul University Faculty of Medicine, Izmir, Turkey; ^2^Department of Internal Medicine and Medical Intensive Care, Dokuz Eylul University Faculty of Medicine, Izmir, Turkey

## Abstract

**Objective:**

Oxygen therapy is one of the most common treatment modalities for hypoxemic patients, but target goals for normoxemia are not clearly defined. Therefore, iatrogenic hyperoxia is a very common situation. The results from the recent clinical researches about hyperoxia indicate that hyperoxia can be related to worse outcomes than expected in some critically ill patients. According to our literature knowledge, there are not any reports researching the effect of hyperoxia on clinical course of patients who are not treated with invasive mechanical ventilation. In this study, we aimed to determine the effect of hyperoxia on mortality, and length of stay and also possible side effects of hyperoxia on the patients who are treated with oxygen by noninvasive devices.

**Materials and Methods:**

One hundred and eighty-seven patients who met inclusion criteria, treated in Dokuz Eylul University Medical Intensive Care Unit between January 1, 2016, and October 31, 2018, were examined retrospectively. These patients' demographic data, oxygen saturation (SpO_2_) values for the first 24 hours, APACHE II (Acute Physiology and Chronic Health Evaluation II) scores, whether they needed intubation, if they did how many days they got ventilated, length of stay in intensive care unit and hospital, maximum PaO_2_ values of the first day, oxygen treatment method of the first 24 hours, and the rates of mortality were recorded.

**Results:**

Hyperoxemia was determined in 62 of 187 patients who were not treated with invasive mechanic ventilation in the first 24 hours of admission. Upon further investigation of the relation between comorbid situations and hyperoxia, hyperoxia frequency in patients with COPD was detected to be statistically low (16% vs. 35%, *p* < 0.008). Hospital mortality was significantly high (51.6% vs. 35.2%, *p* < 0.04) in patients with hyperoxia. When the types of oxygen support therapies were investigated, hyperoxia frequency was found higher in patients treated with supplemental oxygen (nasal cannula, oronasal mask, high flow oxygen therapy) than patients treated with NIMV (44.2% vs. 25.5%, *p* < 0.008). After exclusion of 56 patients who were intubated and treated with invasive mechanical ventilation after the first 24 hours, hyperoxemia was determined in 46 of 131 patients. Mortality in patients with hyperoxemia who were not treated with invasive mechanical ventilation during hospital stay was statistically higher when compared to normoxemic patients (41.3% vs 15.3%, *p* < 0.001).

**Conclusion:**

We report that hyperoxemia increases the hospital mortality in patients treated with noninvasive respiratory support. At the same time, we determined that hyperoxemia frequency was lower in COPD patients and the ones treated with NIMV. Conservative oxygen therapy strategy can be suggested to decrease the hyperoxia prevalence and mortality rates.

## 1. Introduction

Hypoxemia in critically ill patients is a frequent finding independent of the underlying disease state. Hypoxemia is a severe challenge that initiates cellular damage and multiple organ failure via increasing respiratory and cardiovascular work load and affecting hematological system. Therefore, oxygen treatment is obligatory during hypoxemia state. Oxygen (O_2_) treatment can be defined simply as applying higher concentrations of oxygen than room air. Although the most frequent indication of oxygen treatment is preventing and treating hypoxemia, the main goal is ameliorating tissue hypoxia. Oxygen treatment, likewise all other treatment modalities, should be applied to appropriate patient on the right time, in appropriate doses, and the benefits or consequences like oxygen toxicity should be taken into account.

Although guidelines recommend oxygen treatment in the management of critically ill patients, there are no cutoff limits for the target partial oxygen pressure (PaO_2_) or oxygen saturation (SpO_2_) [1–3]. Therefore, inappropriate oxygen treatment may give rise to iatrogenic hyperoxia and unfavorable results [4]. Potential effects of high concentrations of oxygen include interruptions in normal physiological functions, oxygen related tissue injury/oxygen toxicity, and carbon dioxide retention.

Although hyperoxia and hyperoxemia terms are used interchangeably, as a matter of fact hyperoxia is the exposure of cells, tissues, and organs to high oxygen support and hyperoxemia is PaO_2_ values of higher than 100 mmHg in arterial blood gas analysis. However, clinical studies in the literature do not define a standard value for the limits of hyperoxemia. Various studies report PaO_2_ values ranging between 100 and 487 mmHg and oxygen saturation values higher than %96–98 as hyperoxemia [5, 6].

When “oxygen saturation” is used to define hyperoxemia, the situation seems to be more complicated because oxyhemoglobin dissociation curve is sigmoidal and the measures of oxygen saturation by pulse oximetry have error margins. In oxygen saturation values more than 70%, this error margin is accepted to be approximately ±4-5% due to technical reasons [7].

The pathological effects of hyperoxia on pulmonary physiology includes suppression of respiratory drive to hypoxia, pulmonary vasodilatation, ventilation/perfusion disequilibrium, hypercapnia, atelectasis, acute tracheobronchitis, diffuse alveolar damage, acute respiratory distress syndrome, and bronchopulmonary dysplasia. Main effects of hyperoxia on extrapulmonary organ systems are suppression of erythropoiesis, systemic vasoconstriction, and depression of cardiac output. Tissue damage caused by oxygen toxicity is associated with free oxygen radicals which is an end-product of high oxygen concentration. It was shown in animal models that high oxygen concentrations cause increased production of reactive oxygen end-products. Recent clinical studies report that hyperoxemia in critically ill patients is associated with worse outcome [8].

Almost all of the studies on effects of hyperoxia are conducted on patients treated with invasive mechanical ventilation. According to our current knowledge, there is no clinical study on the effects of hyperoxia in patients treated with noninvasive mechanical ventilation. Therefore, we decided to study the effects of hyperoxemia on patients treated with noninvasive respiratory support.

## 2. Materials and Methods

The study was designed as retrospective cohort study and performed in Department of Medical Intensive Care Unit, Dokuz Eylul University Hospital, Izmir, Turkey, between January 1, 2016, and October 31, 2018. After ethical approval, the database and medical records were screened for the admission.

The need for informed consent was waived because of the study design. The inclusion criteria were as follows: (i) adult patients aged ≥18 years and (ii) patients treated with oxygen via noninvasive methods such as nasal cannula/mask, noninvasive mechanical ventilation (NIMV), or high flow nasal oxygen (HFNO) for more than 24 hours. Patients treated with invasive mechanical ventilation (IMV) since their hospitalization or switched from NIMV to IMV during the first 24 hours, patients receiving vasopressor support or patients who had an ICU stay less than 24 hours were excluded from the study. 227 patients were detected to fulfill the inclusion criteria. Upon careful review of the medical records, 40 of these patients were detected to have missing data and remaining 187 were included for further evaluation.

Age, gender, primary diagnosis, comorbid diseases, APACHE II (Acute Physiology and Chronic Health Evaluation II) scores, intubation during ICU stay, mechanical ventilation days of intubated patients, ICU stay, hospital stay, highest PaO_2_ in the first 24 hours of ICU stay, hourly oxygen saturation levels, oxygen treatment modalities during the first 24 hours of ICU stay, and mortality ratios were recorded.

There is no defined standard value of hyperoxemia depending on previous studies. Some studies used SpO_2_ values whereas some used PaO_2_ to define hyperoxemia. We decided to use SpO_2_ values to define hyperoxemia because we were able to reach records of hourly oxygen saturation values but arterial blood gas analysis including PaO_2_ were measured 2–4 times daily. We decided to use SpO_2_ ≥96% to define hyperoxemia as most of the past studies did [6].

### 2.1. Statistics

All categorical variables are expressed as numbers and percentages and continuous variables were expressed as median and interquartile range (IQR). Categorical variables between groups were compared with chi-square; continuous variables were compared with Student's *t*-test. A two-tailed *p* value of <0.05 was considered statistically significant. Statistical analysis was performed with SPSS (SPSS PC Ver.24; IBM© SPSS Inc., New York, USA).

## 3. Results

Study population consisted of 99 (52.9%) females and 88 (47.1%) males. 112 (59.9%) patients were transferred from Emergency room while 75 (40.1%) patients were transferred from in-hospital services. Mean age was 68.5 ± 15.7. Mean APACHE II scores were calculated as 19.1 ± 6.6. The average of maximum PaO_2_ measured during the first 24 hours of ICU stay was 111.1 ± 50.0 mmHg. 56 (29.9%) patients required IMV after the first 24 hours of ICU stay. Mean duration of mechanical ventilation in these patients was 21.8 ± 55.3 days. Mean durations of ICU and hospital stay were 12.4 ± 23.6 and 30.5 ± 36.4, respectively. Mean of hourly oxygen saturation levels were 94.5 ± 2.8%. Chronic obstructive pulmonary disease, hypertension, and diabetes mellitus were the most common associated comorbidities. Medical records including patient characteristics are shown in Table 1.

In the noninvasive treatment group, 77 (41.2%) patients were treated with oxygen via nasal cannula or mask, venturi mask, and HFNO and 110 (58.8%) patients were treated with NIMV. Out of 187 patients, 56 (29.9%) died in ICU, 20 (10.7%) died during follow-up in the other units after ICU discharge, and total in-hospital mortality was calculated as 40.6%.

We detected 62 patients to be exposed to hyperoxia (SaO_2_ ≥96%) when we evaluated mean oxygen saturations in the first 24 hours of ICU stay. Mean oxygen saturation of the remaining 125 patients was less than 96%. There was no statistically significant difference among hyperoxic and normoxic groups regarding mean age, male/female ratio, APACHE II scores, IMV days, and ICU and hospital stay (Table 2). Mean PaO_2_ values in the first 24 hours of ICU stay were significantly higher in hyperoxic group (131.1 ± 55.5 vs 101.2 ± 43.9, *p* < 0.001). Analysis of association of comorbid diseases and hyperoxia revealed that in COPD patients hyperoxia frequency was less (16% vs 35%, *p* < 0.02) but in chronic liver disease patients it was more frequent (13% vs 4%, *p* < 0.04).

There was no statistically significant difference in indication for invasive mechanical ventilation and ICU mortality between two groups, but hospital mortality was significantly higher in the hyperoxia group (51.6% vs 35.2%, *p* < 0.04). Further analysis of the oxygen treatment modalities detected higher rates of hyperoxia in patients treated with oxygen therapy (nasal/mask, HFNO) when compared with patients treated with NIMV (44.2% vs 25.5%, *p* < 0.008) (Table 2).

56 patients out of 187 in the study population experienced worsening in respiratory failure after 24 hours of ICU stay and had to be intubated and invasively ventilated whereas 131 patients did not require any invasive support. Hyperoxia in the noninvasively treated group was observed in 46 (35.1%) patients (Table 3).

Mean PaO_2_ values of the patients treated noninvasively in the first 24 hours of ICU stay were detected to be significantly higher in hyperoxic group when compared to normoxic patients (134.2 ± 61.5 mmHg vs 100.6 ± 44.6 mmHg, *p* < 0.001). Frequency of hyperoxia was significantly less in COPD patients when compared to other comorbidities (15.2% vs 38.8%, *p* < 0.01).

Although ICU mortality in noninvasively treated patients was detected to be higher in hyperoxic group, this did not reach a statistical significance (15.2% vs 9.4%, *p* > 0.05). On the other hand, hospital mortality was significantly higher in hyperoxic group (45.3% vs 15.3%, *p* < 0.001). Hyperoxia rate was significantly higher in patients treated with supplemental oxygen when compared to the ones treated with NIMV (48.1% vs 26.6%, *p* < 0.001) (Table 3).

## 4. Discussion

Oxygen treatment is one of the most common applied treatment modalities in ICU. In the past, it was believed that hypoxemia is harmful and mild/moderate hyperoxemia is beneficial for living organisms. Recent studies reporting negative effects of hyperoxemia draw attention on this topic again. Oxygen is needed to be applied to the right patient on the right time and at appropriate doses. It is already known that unnecessary overtreatment with oxygen results in hyperoxia and oxygen toxicity [9].

When we evaluated studies associated with hyperoxia we realised that vast majority of the study population consisted of patients treated with IMV. General principle in avoiding oxygen toxicity in invasively ventilated patients is to reduce oxygen supply to 60% and lower as soon as possible. Hyperoxia may be a challenge in patients suffering from respiratory failure but are noninvasively ventilated. Therefore, we aimed to study the rate and negative outcome effects of hyperoxia in patients who received at least 24 hours of noninvasive oxygen support.

Although there is conflicting data about the risks of hyperoxia in ICU patients, recent clinical studies point out that hyperoxia may be associated with worse outcome in some critically ill patients [8]. Hyperoxia is common in especially mechanically ventilated patients. The incidence in this population varies between 16 and 50% [10]. Observational cohort studies report that hyperoxia is identified in more than 50% of the patients that are mechanically ventilated in the first 24 hours of ICU stay [11, 12]. Although two meta-analyses report that hyperoxia is associated with increased mortality in different subgroups of ICU patients, the high heterogeneity of studies produce conflict to reach a statement [13, 14].

There is no standard limit to define hyperoxia in the past studies. Different studies used SpO_2_ or PaO_2_ to define hyperoxia. PaO_2_ limits to define hyperoxemia varied in a wide range from 100 to 487 mmHg. The same is observed in studies using SpO_2_. In a study by Suzuki et al., hyperoxemia was defined as SpO_2_ >%98 and FiO_2_, SpO_2_, and PaO_2_ values were recorded quarterly. This study aimed to identify at which level of FiO_2_ negative effects of hyperoxia started [10]. De Jonge et al. calculated mean FiO_2_ and PaO_2_ of patients treated with IMV in the first 24 hours of ICU stay and reported that high FiO_2_ values (with accordingly low or high PaO_2_ values) were independently associated with in-hospital mortality [11]. Girardis et al. conducted a randomised controlled study to define the impact of conservative (PaO_2_: 70–100 mmHg or SpO_2_: 94–98%) and conventional (PaO_2_ <150 mmHg or SpO_2_: 97–100%) oxygen treatment on mortality in ICU patients. Mortality was significantly lower in the conservative group. Besides less shock, liver failure and episodes of bacteremia were reported in the conservative group [8]. Six et al. studied the relation of hyperoxia with ventilator associated pneumonia (VAP), APACHE II score, blood transfusion, and proton pump inhibitor consumption in ICU patients treated with IMV for more than 48 hours. In this study, hyperoxemia was defined as PaO_2_ >120 mmHg and included all patients displaying PaO_2_ >120 mmHg in at least one arterial blood gas analysis. Hyperoxemia was reported to be an independent factor for VAP [15].

While planning our retrospective study, we evaluated all arterial blood gas analysis results and medical records including hourly oxygen saturation. We decided to use mean oxygen saturation values to define hyperoxemia because we detected 2 to 4 arterial blood gas analyses for each patient which would not represent a 24-hour follow-up.

In our study, COPD patients in hyperoxia group were less than normoxia group. This may have a reasonable explanation. Usually, health workers avoid applying high concentrations of oxygen in COPD patients because it is widely known to cause carbon dioxide retention by respiratory depression. We also detected less hyperoxemia in chronic liver failure patients but total chronic liver failure patients were few to conclude a statement.

When recent studies on hyperoxemia are evaluated, it is seen that most of them are conducted on different disease subgroups. Negative effects of hyperoxemia on mortality have been reported in resuscitated patients due to cardiac arrest [16] and acute ischemic stroke patients [6, 13, 14]. Another study by Ihle et al. reported no association between hyperoxia in the first 24 hours of ICU stay and mortality in out-hospital cardiac arrest cases [17]. An analysis of these studies shows us that vast majority of them are conducted on invasively ventilated patients since the first day of ICU stay, but we excluded invasively ventilated patients. We included critically ill patients who were treated with oxygen supply or NIMV during the first 24 hours of ICU stay. According to our up-to-date knowledge, there is no study on the effects of hyperoxia in these patients. Although there was no significant difference in ICU mortality, we detected significantly increased in-hospital mortality in hyperoxic group. Eastwood et al. reported no association between hyperoxia and mortality in invasively ventilated ICU patients during the first 24 hours. But they used the highest alveolar-arterial oxygen gradient (A-a) and the worst arterial blood gas analysis data in the first 24 hours instead of mean values which may not reflect the degree of hyperoxemia in these patients [12]. Different study reports evaluating effects of hyperoxia in the early hospitalization period on mortality give rise to the need of randomised prospective studies with more homogeneous study populations. Although none of the 187 patients included in our study were treated with IMV during the first 24 hours, 56 of them required intubation afterwards. Further analysis of the remaining 131 patients detected that hyperoxemia during the first 24 hours of ICU stay significantly increased mortality. According to our knowledge, this is the first study demonstrating impact of hyperoxia on mortality in patients treated with supplemental oxygen or noninvasive ventilation.

There are also technical differences between different noninvasive oxygen treatment modalities. In NIMV and new generation HFNO devices oxygen concentration can be adjusted more accurately but in conventional oxygen treatment modalities like nasal cannula, mask, or old-generation HFNO devices patients are more vulnerable to hyperoxia.

Panwar et al. in a multicentered, randomised controlled study compared conservative (SpO_2_: 88–92%) and liberal (SpO_2_ > 96%) oxygen treatment goals in patients predicted to receive IMV for more than 24 hours. As expected, hyperoxia was more frequent in liberal group. There was no significant difference between two groups in development of new organ failure, ICU or 90-day mortality [6]. Although it is known that usually a SpO_2_ of 94–98% or PaO_2_ of 88–100 mmHg is sufficient, traditional oxygen treatment is generally more liberal and hyperoxia is inevitable. Although liberal oxygen treatment is a security against hypoxia, a more conservative approach may prevent hyperoxia and its potential harmful effects. The limitations of our study are as follows. Firstly, it is a retrospective cohort study. Secondly, the study population could be larger probably affecting the ICU mortality rates. Since it was designed retrospectively we could not include more patients because of missing data. Although the study population was different from the prior studies on hyperoxemia we could not use mean, highest or lowest PaO2 levels because of limited daily data

## 5. Conclusion

Hyperoxia increases in-hospital mortality in patients treated with noninvasive oxygen. Additionally, the rate of hyperoxia is less in patients treated with NIMV and COPD patients. Likewise, in COPD patients, applying oxygen conservatively will reduce hyperoxia rates. We studied the effects of hyperoxia in medical ICU patients. To define the effects of hyperoxia on specific patient groups, more effort is needed. Also, the clinical effect of prospectively determined oxygenation goals is another hot topic to be studied.

## Figures and Tables

**Table 1:** Patient characteristics.

Age (years)	68.5 ± 15.7
APACHE II	19.1 ± 6.6
Highest PaO_2_ during the first 24 hours (mmHg)	111.1 ± 50.0
Requirement for IMV, (*n*, %)	56 (29.9)
Days on IMV (days)	21.8 ± 55.3
ICU stay (days)	12.4 ± 23.6
Hospital stay (days)	30.5 ± 36.4
Mean SaO_2_ during the first 24 hours (%)	94.5 ± 2.8
Gender (m/f)	88/99
Transferred from (*n*, %)	
Emergency service	112 (59.9)
Other in-hospital	75 (40.1)
Comorbidities (*n*, %)	
COPD	54 (28.9)
CHD	39 (20.9)
CHF	39 (21.4)
CRF	32 (17.1)
HT	101 (54)
DM	59 (31.6)
CLF	13 (7)
Malignancy	54 (29.9)
Dementia	8 (4.3)
O_2_ treatment modality in the first 24 hours (*n*, %)	
Oxygen supply (nasal mask and HFOT)	77 (41.2)
NIMV	110 (58.8)
Outcome *(n*, %)	
Died	56 (29.9)
Discharged	131 (70.1)
Hospital mortality (*n*, %)	76 (40.6)

COPD: chronic obstructive pulmonary disease, CHD: coronary heart disease, CHF: congestive heart failure, CRF: chronic renal failure, HT: hypertension, DM: diabetes mellitus, and CLF: chronic liver failure.

**Table 2:** Hyperoxic and normoxic patient profiles.

	Hyperoxia group *n* = 62	Normoxia group *n* = 125	*p* value
Age (years)	68.3 ± 17.5	68.6 ± 14.8	>0.05
APACHE II	15.7 ± 6.7	16.3 ± 6.6	>0.05
Highest PaO_2_ (mmHg)	131.1 ± 55.5	101.2 ± 43.9	<0.001
IMV days (*n* = 24/48)	13.5 ± 41.1	25.9 ± 61.1	>0.05
ICU stay (days)	11.8 ± 25.8	12.7 ± 22.6	>0.05
Hospital stay (days)	33.6 ± 42.3	28.9 ± 33.1	>0.05
Gender (f/m)	36/26	63/62	>0.05
Comorbidities (*n*, %)			
COPD	10 (16.1)	44 (35.2)	<0.02
CHD	12 (19.4)	27 (21.6)	>0.05
CHF	12 (19.4)	28 (22.4)	>0.05
CRF	12 (19.4)	20 (16.0)	>0.05
HT	31 (50.0)	70 (56.0)	>0.05
DM	17 (27.4)	42 (33.6)	>0.05
CLF	8 (12.9)	5 (4.0)	<0.04
Malignancy	22 (35.5)	34 (27.2)	>0.05
Dementia	4 (6.5)	4 (3.2)	>0.05
IMV required (*n*, %)	24 (38.7)	48 (38.4)	>0.05
ICU mortality (*n*, %)	18 (29.0)	38 (30.4)	>0.05
Hospital mortality (*n*, %)	32 (51.6)	44 (35.2)	<0.04
O_2_ treatment modality (*n*, %)			
Oxygen supply/NIMV	34/28	43/82	<0.008

COPD: chronic obstructive pulmonary disease, CHD: coronary heart disease, CHF: congestive heart failure, CRF: chronic renal failure, HT: hypertension, DM: diabetes mellitus, and CLF: chronic liver failure.

**Table 3:** Patient profiles treated only noninvasively.

	Hyperoxia group *n* = 46	Normoxia group *n* = 85	*p* value
Age (years)	69.1 ± 17.3	68.3 ± 14.9	>0.05
APACHE II	15.0 ± 6.6	15.0 ± 6.1	>0.05
Highest PaO_2_ (mmHg)	134.2 ± 61.5	100.6 ± 44.6	<0.001
ICU stay (days)	7.2 ± 5.4	6.2 ± 4.3	>0.05
Hospital stay (days)	30.9 ± 38.4	23.2 ± 20.6	>0.05
Gender (f/m)	23/23	46/39	>0.05
Comorbidities (*n*, %)			
COPD	7 (15.2)	33 (38.8)	<0.01
CHD	10 (21.7)	15 (17.6)	>0.05
CHF	11 (23.9)	19 (22.4)	>0.05
CRF	9 (19.6)	12 (14.1)	>0.05
HT	25 (54.3)	51 (60.0)	>0.05
DM	13 (28.3)	29 (34.1)	>0.05
CLF	7 (15.2)	4 (4.7)	>0.05
Malignancy	15 (32.6)	21 (24.7)	>0.05
Dementia	3 (6.5)	3 (3.5)	>0.05
ICU mortality (*n*, %)	7 (15.2)	8 (9.4)	>0.05
Hospital mortality (*n*, %)	19 (41.3)	13 (15.3)	<0.001
O_2_ treatment modality (*n*, %)			
Supplemental oxygen/NIMV	25/21	27/58	<0.02

COPD: chronic obstructive pulmonary disease, CHD: coronary heart disease, CHF: congestive heart failure, CRF: chronic renal failure, HT: hypertension, DM: diabetes mellitus, and CLF: chronic liver failure.

## Data Availability

The data used to support the findings of this study were supplied by Dokuz Eylul University Hospital Patient Records under license and so cannot be made freely available. Requests for access to these data should be made to Aysun Genc by contacting aysun.genc@deu.edu.tr or sistem.analiz@deu.edu.tr.
